# The Effect of Cerebellar tDCS on Sequential Motor Response Selection

**DOI:** 10.1007/s12311-019-01029-1

**Published:** 2019-05-06

**Authors:** Bryant J. Jongkees, Maarten A. Immink, Olga D. Boer, Fatemeh Yavari, Michael A. Nitsche, Lorenza S. Colzato

**Affiliations:** 1 Cognitive Psychology Unit & Leiden Institute for Brain and Cognition, Leiden University, Wassenaarseweg 52, 2333 AK Leiden, The Netherlands; 2 School of Health Sciences & Cognitive Neuroscience Laboratory, University of South Australia, Adelaide, Australia; 3 Leibniz Research Centre for Working Environment and Human Factors, Technical University Dortmund, Dortmund, Germany; 4 Department of Clinical Neurophysiology, Georg-August University Göttingen, Göttingen, Germany; 5 Department of Neurology, University Medical Hospital Bergmannsheil, Bochum, Germany; 6 Department of Cognitive Psychology & Institute of Cognitive Neuroscience, Faculty of Psychology, Ruhr University Bochum, Bochum, Germany; 7 Institute for Sports and Sport Science, University of Kassel, Kassel, Germany

**Keywords:** Cerebellum, Transcranial direct current stimulation, Response selection, Sequence learning, Serial reaction time task

## Abstract

Transcranial direct current stimulation (tDCS) transiently alters cortical excitability and synaptic plasticity. So far, few studies have investigated the behavioral effects of applying tDCS to the cerebellum. Given the cerebellum’s inhibitory effects on cortical motor areas as well as its role in fine motor control and motor coordination, we investigated whether cerebellar tDCS can modulate response selection processes and motor sequence learning. Seventy-two participants received either cerebellar anodal (excitatory), cathodal (inhibitory), or sham (placebo) tDCS while performing a serial reaction time task (SRTT). To compare acute and long-term effects of stimulation on behavioral performance, participants came back for follow-up testing at 24 h after stimulation. Results indicated no group differences in performance prior to tDCS. During stimulation, tDCS did not affect sequence-specific learning, but anodal as compared to cathodal and sham stimulations did modulate response selection processes. Specifically, anodal tDCS increased response latencies independent of whether a trained or transfer sequence was being performed, although this effect became smaller throughout training. At the 24-h follow-up, the group that previously received anodal tDCS again demonstrated increased response latencies, but only when the previously trained sequence and a transfer sequence had to be performed in the same experimental block. This increased behavioral interference tentatively points to a detrimental effect of anodal cerebellar tDCS on sequence consolidation/retention. These results are consistent with the notion that the cerebellum exerts an inhibitory effect on cortical motor areas, which can impair sequential response selection when this inhibition is strengthened by tDCS.

## Introduction

Recent years have seen a substantially growing interest in non-invasive methods of brain stimulation. In particular, transcranial direct current stimulation (tDCS) has received considerable attention as a means to transiently alter cortical excitability and synaptic plasticity [1–4]. Although many studies have examined the behavioral and physiological effects of stimulating cortical areas such as the dorsolateral prefrontal cortex and primary motor area (M1), only recently have studies begun to investigate the cerebellum as a potential site of stimulation [5–8]. The cerebellum plays a critical role in sensorimotor control, such as planning, organization, and initiation of movement [9]. Considering the importance of response selection in motor sequence learning [10–12], we thus hypothesized that cerebellar tDCS could modulate motor response selection and serial sequence learning. However, previous literature gives rise to opposite hypotheses regarding the effects of cerebellar tDCS on response selection and sequence learning. Therefore, in the present study, we set out to clarify the effects of cerebellar tDCS on motor response selection under sequential learning conditions using a version of the serial reaction time task (SRTT) [13].

tDCS is typically applied by mounting two electrodes on the scalp, with a constant current of 1–2 mA running between the electrodes. This alters the excitability and spontaneous activity of neuronal populations in a polarity-dependent manner: typically, neurons beneath the anode show enhanced excitability and thus have an increased likelihood of firing due to a subthreshold depolarization of the resting membrane potential, whereas neurons beneath the cathode are slightly hyperpolarized and thus have a reduced likelihood of firing [4]. At longer stimulation periods, tDCS can also affect neural plasticity for minutes or hours following stimulation [2, 3, 14] by producing changes in levels of glutamate and GABA [15–18].

Previous research on cerebellar tDCS has shown that anodal relative to cathodal and sham tDCS applied over the cerebellum can enhance learning in motor adaptation tasks, specifically by increasing online rather than offline learning [19]. Along the same lines, it was shown that older participants who received anodal tDCS demonstrated enhanced performance in a motor adaptation task with a rate that was similar to younger subjects [20]. Further, stimulating the cerebellum via tDCS seemed to enhance locomotor adaptation [21] and visuomotor adaptation but not intermanual transfer of learning [22]. Although these studies focused on learning within the domain of motor adaptation rather than motor sequence learning, these different forms of learning are known to rely on shared neurobiological systems, i.e., cerebello-cortical and cortico-striatal networks [23]. The aforementioned findings suggest that cerebellar tDCS could enhance sequential motor learning as well.

Two reports indicate that, indeed, motor sequence learning can be enhanced by anodal cerebellar tDCS [24, 25]. In these studies, participants completed an SRTT including blocks of random responses and blocks including a repeating sequence of responses. Using either a spatially aligned [24] or symbolic [25] stimulus–response mapping, both studies showed that anodal stimulation of the cerebellum increased the difference in reaction time (RT) between random and sequenced response blocks, which indicates increased reliance on sequence structure and is typically taken to reflect motor sequence learning.

However, these studies have left open a number of key questions that require further research. By reporting only on the performance difference between random and sequenced blocks, it remains unclear whether cerebellar stimulation affected RT specifically for random responses or sequenced responses, or both. This leaves open the possibility that an increased difference between these types of responses is driven primarily by slower *random* responses rather than faster *sequenced* responses. While such a pattern could still be argued to reflect increased reliance on the trained sequence, it would not be the same as showing that tDCS enhanced the acquisition and execution of trained sequenced responses. The interpretation of these findings is further complicated by the fact that one of the studies reports significant performance improvement on sequenced blocks in the anodal but not in the sham condition [24]. Considering that sequence learning is expected to take place in the absence of stimulation (compare [10, 26, 27]), in this case, the effectiveness of cerebellar tDCS appears to be driven by a lack of improvement in the sham condition rather than enhanced performance due to stimulation. As such, this finding does not unequivocally support an enhancing effect of cerebellar tDCS on motor sequence learning.

The other study [25] does report significant performance improvement under both anodal and sham stimulations, but with greater reduction of error rates from the beginning to end of the SRTT being observed under anodal tDCS. However, this study used a symbolic rather than spatial stimulus–response mapping. Because stimulus–response learning is of critical importance to the acquisition of serial motor sequences [11, 12], the enhanced SRTT performance in this study may have been driven primarily by a facilitated acquisition of abstract stimulus–response associations. Consequently, it is unclear whether the same effect is observable with spatially corresponding stimulus–response associations, which are typically used in SRTT paradigms to investigate sequence learning. Considering also the fact that both aforementioned studies investigated solely the effects of anodal (excitatory) rather than cathodal (inhibitory) stimulation of the cerebellum, there is still much uncertainty about the effects of cerebellar tDCS on motor sequence acquisition.

Further increasing the need for research on the effects of cerebellar tDCS on motor behavior are studies showing that anodal stimulation can also impair rather than improve response selection processes. For example, anodal tDCS has previously produced a delay in the initiation of muscle activity [28] and has impaired handwriting legibility with the non-dominant hand [29]. These findings can be understood by considering that cerebellar tDCS modulates a phenomenon referred to as cerebellar–brain inhibition (CBI) in a polarity-dependent manner [8]. That is, Purkinje cells in the cerebellum exert an inhibitory tone over M1 via the dentate-thalamo-cortical pathway [30, 31], and this inhibition is strengthened by anodal tDCS and weakened by cathodal tDCS relative to sham stimulation [32]. Reduced excitability of M1 due to anodal cerebellar tDCS may have accounted for the previous observations of delayed initiation of muscle activity [28] and impaired handwriting legibility with the non-dominant hand [29]. Furthermore, it is worthy of note that motor sequence acquisition is typically associated with increased M1 excitability [33]. This suggests that anodal cerebellar tDCS may impair response selection and sequence learning, whereas these processes might be enhanced under cathodal tDCS via a respective decrease and increase in M1 excitability.

In order to clarify the effects of cerebellar tDCS on response selection and serial sequence learning, the present study assessed SRTT performance with concurrent anodal, cathodal, or sham tDCS of the cerebellum. We included a spatially corresponding stimulus–response mapping to limit potentially confounding effects of stimulation on the learning of abstract stimulus–response associations.

The SRTT is a 4-choice RT task [13] that involves response selection, inhibition of non-target responses, and formation of response sequence structures, each of which may be sensitive to a modulation of M1 excitability via cerebellar tDCS. Typically, a second-order conditional (SOC) response sequence is embedded in the SRTT unbeknownst to the participants. Implicit acquisition of this sequence structure results in increasingly shorter RT and less response errors as the task progresses [13, 26, 27]. However, there is potential difficulty in disentangling the nature of these improvements [34] as performance improvements might not be sequence specific but rather reflect general practice effects [27]. For this reason, a transfer approach is commonly used to judge the extent to which performance improvements rely on the practiced sequence [27, 35, 36]. This was implemented in the present experiment by presenting 10 out of 13 SRTT blocks that exclusively contained the same repeating SOC response sequence. The remaining three blocks (1, 7, and 13) were probe blocks that consisted predominantly of the trained SOC sequence, but also an untrained SOC sequence in order to disentangle sequence-specific learning from general practice effects [27, 36].

To further elucidate the potential behavioral effect of cerebellar tDCS on motor practice, we also applied the learning–performance distinction to our study design. In brief, this concept distinguishes between the short-term change in motor behavior that takes place *during* practice (considered to reflect “performance”) and the resilience of this behavior that is sustained *over time* (considered to reflect “learning”), with the latter arguably being the desired outcome of motor practice [37]. In the context of the present study, this distinction signifies a need to discriminate between potential short-term effects of cerebellar tDCS (as assessed during SRTT training overlapping with stimulation) and long-term effects on consolidation and retention of the motor practice. Underlining the importance of investigating and comparing these effects, previous studies have indicated that cerebellar tDCS primarily influences the immediate performance aspect of motor practice but not its retention [25, 38]. To clarify the potential effect of cerebellar stimulation on consolidation and retention of the practiced motor sequence, in the present study, subjects performed a shortened version of the SRTT at 24-h follow-up without tDCS.

In brief, the present study sets out to investigate the behavioral effects of tDCS on motor response selection and sequence learning. M1 excitability is known to affect these processes [33, 39] and can be indirectly influenced via a strengthening or weakening of CBI using cerebellar tDCS [8, 32]. In light of this indirect effect of cerebellar stimulation on M1 mediated by CBI, we expected anodal relative to sham tDCS to decrease M1 excitability and hinder sequence learning. Behaviorally, this would be reflected in increased overall RT and smaller performance gains throughout practice. In contrast, cathodal relative to sham tDCS was expected to produce the opposite results. Furthermore, to investigate whether cerebellar tDCS affects consolidation and retention of motor practice, we assessed SRTT performance not only during stimulation but also at 24-h follow-up without tDCS.

## Materials and Methods

Informed consent was obtained from all individual participants included in the study. All procedures involving human participants were in accordance with the ethical standards of the institutional and/or national research committee and with the 1964 Helsinki declaration and its later amendments or comparable ethical standards. The protocol was approved by the local ethical committee (Leiden University, Institute for Psychological Research).

### Participants

Seventy-two right-handed, healthy undergraduate students from Leiden University were compensated with partial course credit for participation in a study on brain stimulation. Participants were randomly assigned to receive either anodal (*N* = 24), cathodal (*N* = 24), or sham (*N* = 24) stimulation. Group demographics are presented in Table 1. The groups were comparable with respect to age (*F*[2,69] = .675, *p* = .512), gender distribution (*X*^2^[2, *N* = 72] = .572, *p* = .751), and hours of sleep the night before the experimental sessions (*F*[2, 69] = .118, *p* = .888). Participants were screened individually using the Mini International Neuropsychiatric Interview (MINI), a short, structured interview of approximately 15 min in duration that screens for several psychiatric disorders and drug use [40], and has been used previously in research on tDCS [41] and the SRTT [34, 42]. Participants were included if they met the following criteria: (i) between 18 and 30 years; (ii) no history of neurological or psychiatric disorders; (iii) no history of substance abuse or dependence; (iv) no chronic or acute medication; and (v) no metal implants or cardiac disorders for safety reasons concerning tDCS. Before the start of the study, participants were informed about the procedure and potential side effects of tDCS (i.e., itching, stinging, or burning sensation from the electrodes, reddening of the skin, and headache). None of the participants reported major side effects.

**Table 1:** Group demographics

	Stimulation group		
	Anodal	Cathodal	Sham
Age in years	19.8 (1.6)	19.5 (1.5)	19.3 (1.8)
Gender	F 17, M 7	F 17, M 7	F 19, M 5
Hours of sleep day 1	7.3 (1.8)	7.3 (1.1)	7.6 (1.0)
Hours of sleep day 2	7.4 (1.5)	7.1 (1.2)	7.2 (1.3)

Standard deviation of mean is listed in parentheses

### Cerebellar Transcranial Direction Current Stimulation

Cerebellar tDCS was applied using three electrodes of 35 cm^2^ (5 cm × 7 cm), with the target electrode centered over the inion and the two reference electrodes placed bilaterally over the mastoids to limit the effects of the reference electrodes on cortical activity. Whereas previous studies typically placed the target electrode lateral to the inion to investigate effects of cerebellar stimulation on unimanual performance [24, 25], others have centered the target electrode over the inion for bilateral stimulation of the cerebellum [43–45]. As the SRTT in the present study required bimanual performance, we opted to center the target electrode over the inion. Stimulation consisted of a current of 1 mA delivered by a DC Brain Stimulator Plus (NeuroConn, Ilmenau, Germany), a device complying with the Medical Device Directive of the European Union (CE certified). The current was built up during a fade-in of 10 s, after which stimulation lasted for precisely 20 min and then ended with a 10-s fade-out. Sham stimulation was identical to real stimulation, except for the fact that it lasted for 15 s instead of 20 min; this provides a similar initial sensation as real stimulation but stimulating at such short durations does not produce changes in cortical excitability or plasticity that outlast the intervention [14]. All participants finished the SRTT within the 20 min of stimulation. Impedance was below 15 kΩ throughout the stimulation.

**Fig. 1:**
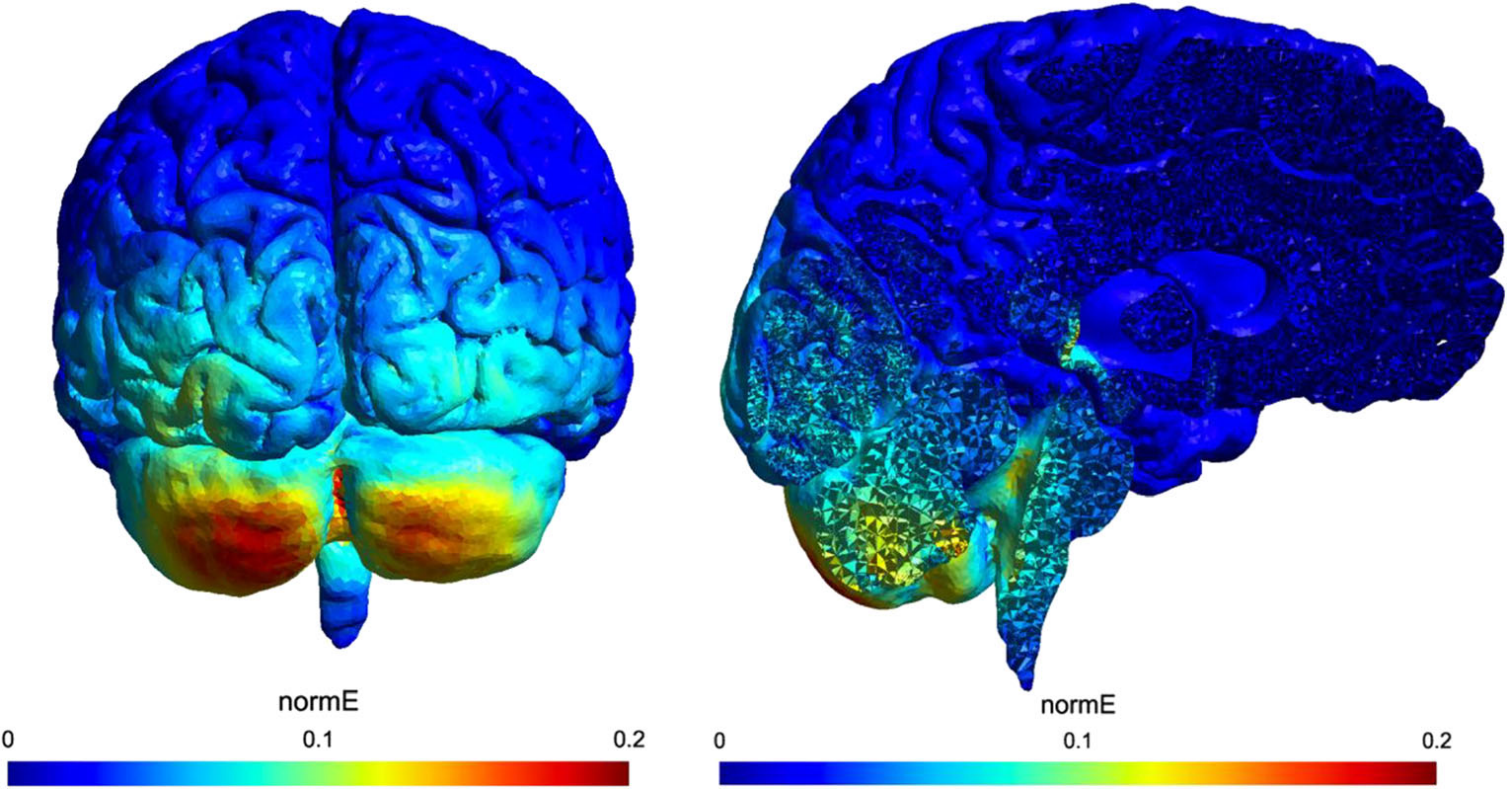
Spatial distribution of the normalized electric field calculated using the SimNIBS pipeline. Anode 5 cm × 7 cm, centered over the inion, 1-mA current; two cathodes over the mastoids, 5 cm × 7 cm, 0.5-mA current each. The average value of EF magnitude in the cerebellar cortex was obtained to be 0.0863 Vm^−1^

Open access SimNIBS software (www.simnibs.org, version 2.0) was used to develop the head model for finite element modeling [46, 47]. SimNIBS uses FreeSurfer and FSL BET to segment the head. The SimNIBS pipeline was applied on a realistic head model (called almi) provided by SimNIBS as an example dataset (available from SimNIBS download section). Five tissue segments are considered in the model: scalp, skull, cerebrospinal fluid, gray matter, and white matter. Electrodes, modeled as saline-soaked 5 × 7 cm^2^ rectangular sponges, were positioned over the inion and mastoids, similar to the experimental montage. Current intensity was set to 1 mA for the electrode over the inion and 0.5 mA for each of the electrodes over the mastoids. The finite element method is employed in SimNIBS to calculate electric field (EF) distribution. The spatial distribution of the norm values of EF is shown in Fig. 1. Electric field is relatively strong at the surface and deep layers of the cerebellum. To calculate the mean value of norm EF in the cerebellar cortex, we used SUIT, a high-resolution atlas template of the human cerebellum and brainstem, which is based on the anatomy of 20 young healthy individuals, in the space defined by the MNI152 template [48]. Cerebellar cortex was extracted from this atlas and registered to the space of MRI data which was used for tDCS modeling. Registration matrix was calculated using FLIRT in FSL 5.0 (http://fsl.fmrib.ox.ac.uk/fsl/fslwiki/).

The potential experience of side effects due to tDCS was assessed through self-report ratings on a 5-point scale for the following: headache, neck pain, nausea, muscle contractions in the face or neck, stinging sensation under the electrodes, burning sensation under the electrodes, and a non-specific, uncomfortable feeling. Consistent with previous studies, the most prominent side effects were stinging and burning sensations under the electrodes [49], although none of the participants voiced major complaints. There were no group differences in terms of self-reported side effects (*p*s > .070 for all group effects). Driven by the behavioral findings reported below, we conducted Bonferonni-corrected post hoc comparisons for the self-reported ratings of the anodal and sham groups, which revealed no group differences for any of the side effects (*p*s > .366). This provides strong evidence that the experience of tDCS side effects does not confound the group differences in terms of SRTT performance reported below.

### Serial Reaction Time Task

To assess motor response selection and sequence learning, participants performed a SRTT [50] conducted through E-Prime 2.0 software (Psychology Software Tools, Inc., Pittsburgh, PA, USA). In this task, four horizontally aligned empty squares are presented in the center of the screen. On each trial, one of the squares turns red and the participant must press a spatially corresponding key on a QWERTY keyboard (from left to right: V, B, N, M) using the index and middle fingers of the left (V, B) and right (N, M) hands. An error sound is presented for 250 ms if the wrong button is pressed, along with the Dutch words *Verkeerde toets*! (Wrong button!) appearing on the screen for 500 ms. If RT exceeds 3000 ms, the Dutch words *Te langzaam*! (Too slow!) are presented on the screen for 500 ms. RT is measured in milliseconds as the latency between stimulus appearance and the respective key press. Following the response, the four empty squares appear for a brief 50-ms response–stimulus interval before a different square turns red. Participants were instructed that accuracy and response speed were equally important in the task.

All participants completed one task familiarization block of 120 randomly sequenced trials prior to stimulation to check for pre-existing group differences in response selection efficiency. Subsequently, tDCS was applied while participants performed 13 training blocks, with each block consisting of 10 cycles of 12 trials. Blocks 2–6 and 8–12 consisted only of the same, repeating 12-item SOC training sequence (i.e., VBVNMBNVMNBM) [51]. Because performance improvement throughout the task may simply reflect a general practice effect rather than sequence-specific learning [27], blocks 1, 7, and 13 were designed to be sequence learning probe blocks. These probe blocks always started and ended with a cycle of the 12-item SOC training sequence. Randomly inserted in the remaining eight cycles of the probe block were two consecutive cycles of an analogous transfer SOC sequence, which was formed by interchanging the V and N keys in the training sequence (i.e., NBNVMBVNMVBM) [52]. As such, the transfer sequence was similar in complexity to the trained SOC sequence. After completion of each block, performance feedback was provided on the monitor to indicate the number of errors and mean RT for the block. This was followed by a 30-s rest interval.

Performance on the transfer sequence offers insight into whether performance improvement throughout the task is specific to the repeating training sequence or whether improvement extends to this untrained transfer sequence. RT is expected to be higher under the less-practiced transfer sequence and larger increases in RT between trained and transfer sequences indicate greater sequence learning.

To assess the potential effect of tDCS on retention of the trained SOC sequence, participants came back to the lab for a 24-h follow-up to complete another three blocks of the SRTT (blocks 14–16) without stimulation. Blocks 14 and 16 were probe blocks organized similarly as probe blocks on the first testing day. Block 15 exclusively contained the trained SOC (as in blocks 2–6 and 8–12 on the first day), to assess long-term retention of the trained sequence without performance interference from having to carry out a less-practiced transfer sequence in the same block.

### Procedure

Upon entering the lab, informed consent was obtained and participants were screened for the inclusion and exclusion criteria for participation in the experiment. Participants who did not meet the criteria were excluded from further participation in the study; those who met all criteria then completed the familiarization block of the SRTT. Subsequently, tDCS electrodes were mounted on the scalp and stimulation was applied for a maximum of 20 min, during which participants completed the 13 training blocks of the SRTT. Stimulation was applied throughout the task, which took no more than 20 min to complete. Immediately after completing the SRTT, the electrodes were removed from the scalp and participants were asked to rate on a 5-point scale to what extent they experienced adverse effects due to the stimulation. None of the participants reported major adverse effects. All participants came back to the lab 24 h after the first session; upon arrival, they completed the follow-up SRTT blocks 14 to 16 without stimulation. Lastly, they were debriefed and thereafter left the lab. The two sessions together took an approximate total of 60 min to complete.

### Analysis

To compare SRTT performance between groups, percent accuracy (ACC) was calculated for each participant during familiarization and training (i.e., during stimulation), and at 24 h follow-up. ACC for each of these three phases was separately submitted to one-way analysis of variance (ANOVA) using the aov function in R, version 3.4.3 [53].

For analysis of RT performance, all incorrect trials were removed (2.80% for familiarization, 3.06% for training, and 2.69% for test). RT data in familiarization, training, and follow-up SRTT phases were analyzed using linear mixed-effects modeling (LMM) with the lme4 package in R [54]. The LMM approach does not require data averaging like traditional ANOVA analysis approaches and so LMM provides a more selective approach to investigating experimental effects and interactions [55]. This is because LMM allows for control of variance associated with random factors [56]. In the present LMM analyses, we treated participants and response stimuli as random factors. For fitted LMM models, we used the car package in R [57] to conduct type III Wald tests with Satterthwaite degrees of freedom approximation [58].

For analysis of data from the first day, LMM for RT in familiarization included group (sham, anodal, cathodal stimulation) as a fixed factor. Thereafter, to evaluate overall performance improvements with the training sequence, LMM for RT during training was conducted on the 10 blocks that involved only the trained SOC sequence (blocks 2–6 and 8–12). For this, we included group and block as fixed factors. Subsequently, to evaluate sequence-specific learning by comparing performance on the trained SOC sequence and the transfer SOC sequence, we conducted LMM on RT in the three probe blocks during training (blocks 1, 7, and 13). Here, we included group, block, and sequence type (trained and transfer SOC) as fixed factors.

To evaluate retention of sequence-specific learning, we compared performance at the end of training relative to performance at 24-h follow-up. First, we conducted LMM on RT in the final probe block of training (block 13) and the two probe blocks at test (blocks 14 and 16) with group, block, and sequence type as fixed factors. Finally, to evaluate RT performance when only the trained SOC sequence was present at follow-up, we conducted LMM on block 15 with group as a fixed factor. Post hoc tests for significant effects and interactions were conducted using multiple comparisons of factor contrasts via the phia R package [59]. Significant effects and interactions from LMM were graphed using the effects [60] and ggplot2 [61] R packages.

## Results

### Reaction Time

#### Familiarization and Training (Day 1)

**Fig. 2:**
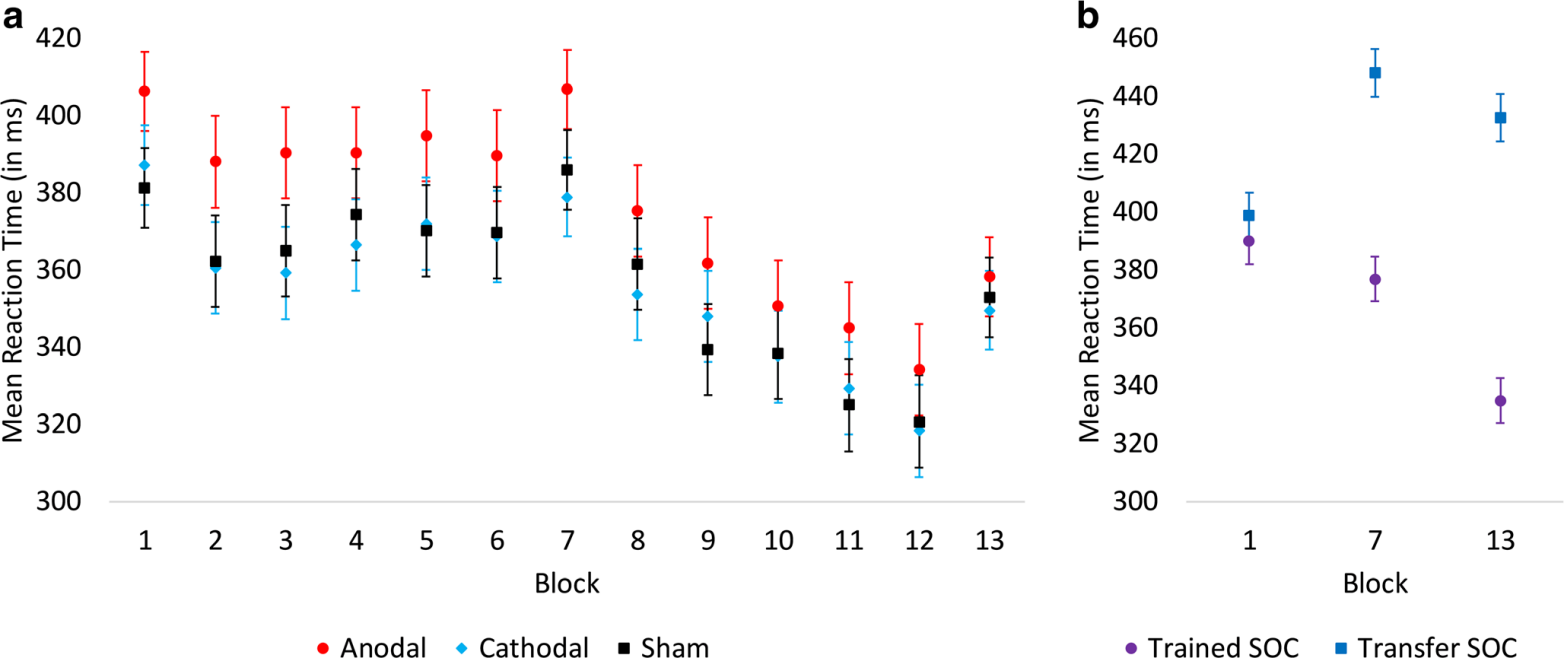
**a** Mean RT (in ms) as a function of stimulation group and blocks 1–13 (i.e., those performed on day 1). The anodal stimulation group demonstrates longer RT in early blocks, but RT no longer differs from the other groups at the end of training. **b** Mean RT (in ms) as a function of sequence type in the three probe blocks on day 1 (blocks 1, 7, and 13). Performance on both sequences is comparable in the first block, but diverges in the second and third probe blocks, demonstrating a typical sequence learning pattern. Error bars represent standard error of the means

At the outset of the experiment, RT performance did not differ significantly between the sham stimulation group (*M* = 432.8 ms, SE = 16.5), the anodal stimulation group (*M* = 450.0 ms, SE = 16.5), and the cathodal stimulation group (*M* = 429.9 ms, SE = 16.5) (*X*^2^[2, *N* = 8640] = 1.66, *p* = .44). For RT in blocks 2–6 and 8–12, in which only the trained SOC sequence was performed, there was a significant group × block interaction (*X*^2^[18, *N* = 83,863] = 55.16, *p* < .001). The source of this interaction was the anodal stimulation group exhibiting longer RT in block 2 than the sham stimulation group (*X*^2^[1, *N* = 5625] = 3.93, *p* < .05) (see Fig. 2a). In addition, the anodal stimulation group had longer RT than the cathodal stimulation group in blocks 2 (*X*^2^[1, *N* = 5625] = 4.42, *p* < .05) and 3 (*X*^2^[1, *N* = 5625] = 4.64). There were no significant differences in RT performance between stimulation groups in the other SOC training blocks. Analysis of RT in the probe blocks revealed a significant block × sequence type interaction (*X*^2^[2, *N* = 25,015] = 147.56, *p* < .001), and a significant group × block interaction (*X*^2^[4, *N* = 25,015] = 24.48, *p* < .001). The significant block × sequence type interaction (see Fig. 2b) indicates a typical sequence learning pattern across groups whereby RT for transfer sequences becomes increasingly longer than RT for trained sequences from block 1 (*X*^2^[1, *N* = 8457] = 7.64, *p* < .01) to block 7 (*X*^2^[1, *N* = 8457] = 483.82, *p* < .001) and block 13 (*X*^2^[1, *N* = 8457] = 896.97, *p* < .001), owing to RT decreases for the trained sequences across probe blocks while RT for the transfer sequence remains relatively unchanged. This indicates selective improvement on the trained but not the transfer sequence. The significant group × block interaction (see Fig. 2a) reveals significantly longer RT for the anodal stimulation group than the sham stimulation group in block 1 (*X*^2^[1, *N* = 5625] = 4.10, *p* < .05). The anodal stimulation group also performed with significantly longer RT than the cathodal stimulation group in block 2 (*X*^2^[1, *N* = 5625] = 5.68, *p* < .05). No other significant differences in RT were observed between groups in the probe blocks. However, tDCS did not affect sequence-specific learning, since in probe blocks, neither the group × sequence type (*X*^2^[2, *N* = 25,015] = .315, *p* = .85) nor group × block × sequence type (*X*^2^[4, *N* = 25,015] = 2.34, *p* = .67) interactions were significant.

#### 24-H Follow-up

**Fig. 3:**
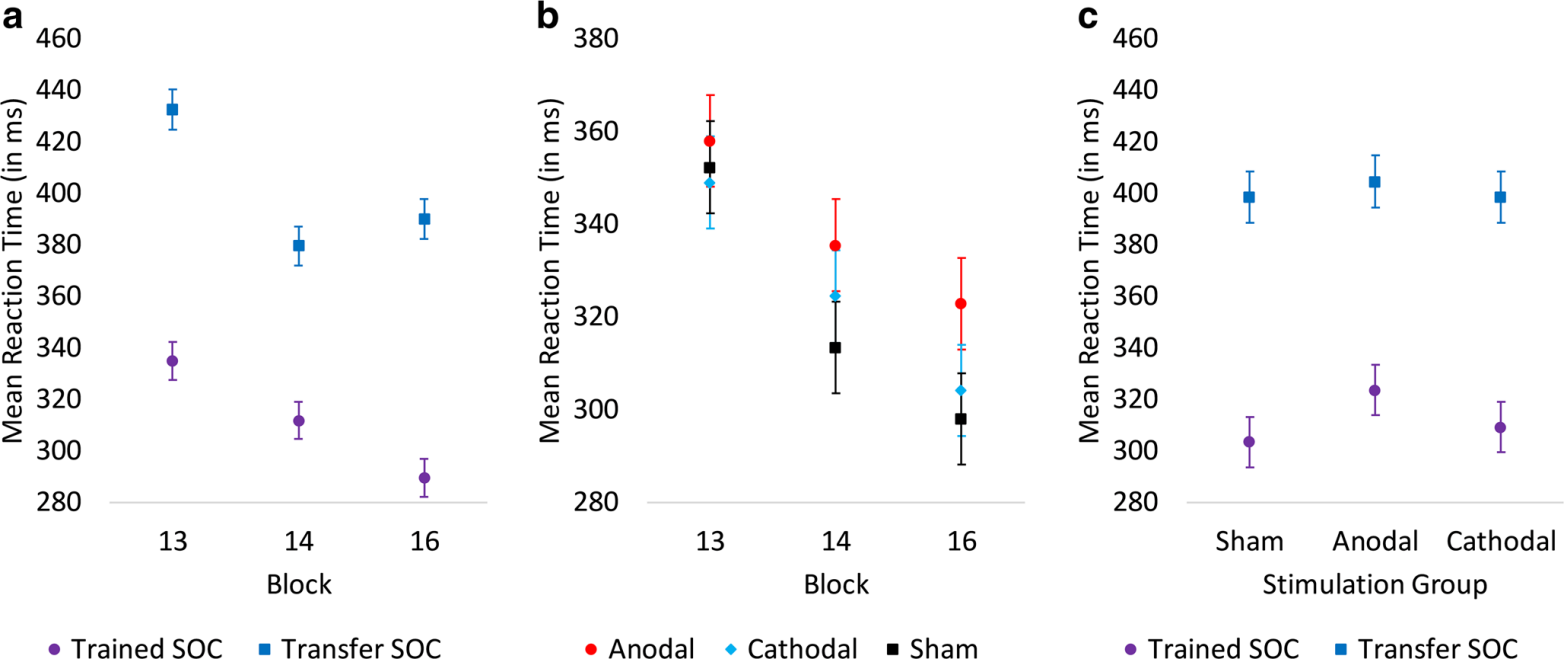
**a** Mean RT (in ms) as a function of sequence type in the third and final probe blocks during training (block 13) and the probe blocks during test (24-h follow-up). Performance of both sequences benefits from overnight sleep, but further exposure to the trained SOC facilitates performance of this sequence, whereas it interferes with performance of the transfer sequence. **b** Mean RT (in ms) as a function of stimulation group in the third and final probe blocks during training (block 13) and the probe blocks during test (24-h follow-up). The groups no longer differed at the end of training on day 1, but the anodal stimulation group again demonstrated longer RT in probe blocks at 24-h follow-up. **c** Mean RT (in ms) as a function of sequence type and stimulation group collapsed across the two probe blocks 14 and 16 at 24-h follow-up. The anodal stimulation group demonstrates longer RT than cathodal and sham groups, and this difference is larger for the trained SOC. Error bars represent standard error of the means

Analysis of RT in the final training probe block (block 13) and the two probe blocks at follow-up (blocks 14 and 16) revealed a significant block × sequence type interaction (*X*^2^[2, *N* = 25,019] = 32.34, *p* < .001). RT for both sequence types decreased from block 13 to block 14, suggesting a general practice effect from the first to the second testing day. From blocks 14 to 16, RT further decreased for the trained sequence but increased for the transfer sequence, reflecting additional sequence-specific learning (see Fig. 3a). In addition, there was a significant interaction between group and block (*X*^2^[4, *N* = 25,019] = 27.45, *p* < .001), and a significant group and sequence type interaction (*X*^2^[2, *N* = 25,019] = 12.81, *p* < .01). The group × block interaction, illustrated in Fig. 3b, is based on all three groups demonstrating decreased RT between blocks 13 and 14 (*p* < .001), while only the cathodal group demonstrated a significant decrease in RT between blocks 14 and 16 (*X*^2^[1, *N* = 5592] = 10.71, *p* < .01).

All three groups demonstrated significantly longer RT in transfer sequence than the trained sequence (all *p* < .0001). However, the significant group × sequence type interaction appeared to be based on a larger RT difference for the trained sequence between anodal and sham groups (13.0 ms slower for anodal group) as compared to anodal and cathodal (6.1 ms slower for anodal group) as well as cathodal and sham group (6.8 ms slower for cathodal group) comparisons (see Fig. 3c). None of these group differences reached significance (*p* = .15, .50, and .44, respectively). For transfer sequence RT, non-significant (*p* > .81) differences were smaller when comparing the anodal group to cathodal (2.24 ms slower for cathodal) and sham (1.27 ms slower for sham) groups.

No significant group differences were observed for RT in block 15, which involved only the trained sequence (*X*^2^[2, *N* = 8491] = 4.05, *p* = .13).

#### Accuracy

At familiarization, we observed no significant differences in ACC between the anodal group (*M* = 97.3%, SD = 2.4%), cathodal group (*M* = 96.9%, SD = 2.9%), and sham group (*M* = 97.5%, SD = 2.2%) (*F*[2,69] = .34, *p* = .71). Similarly, during training on day 1, there was no effect of group on ACC, nor any interaction with block and/or sequence type (all *F*s < 1.15 and all *p*s > .36). At follow-up on day 2, there were also no group differences in block 15, which involved only the trained sequence (*F*[2,69] = 1.0, *p* = .37).

**Fig. 4:**
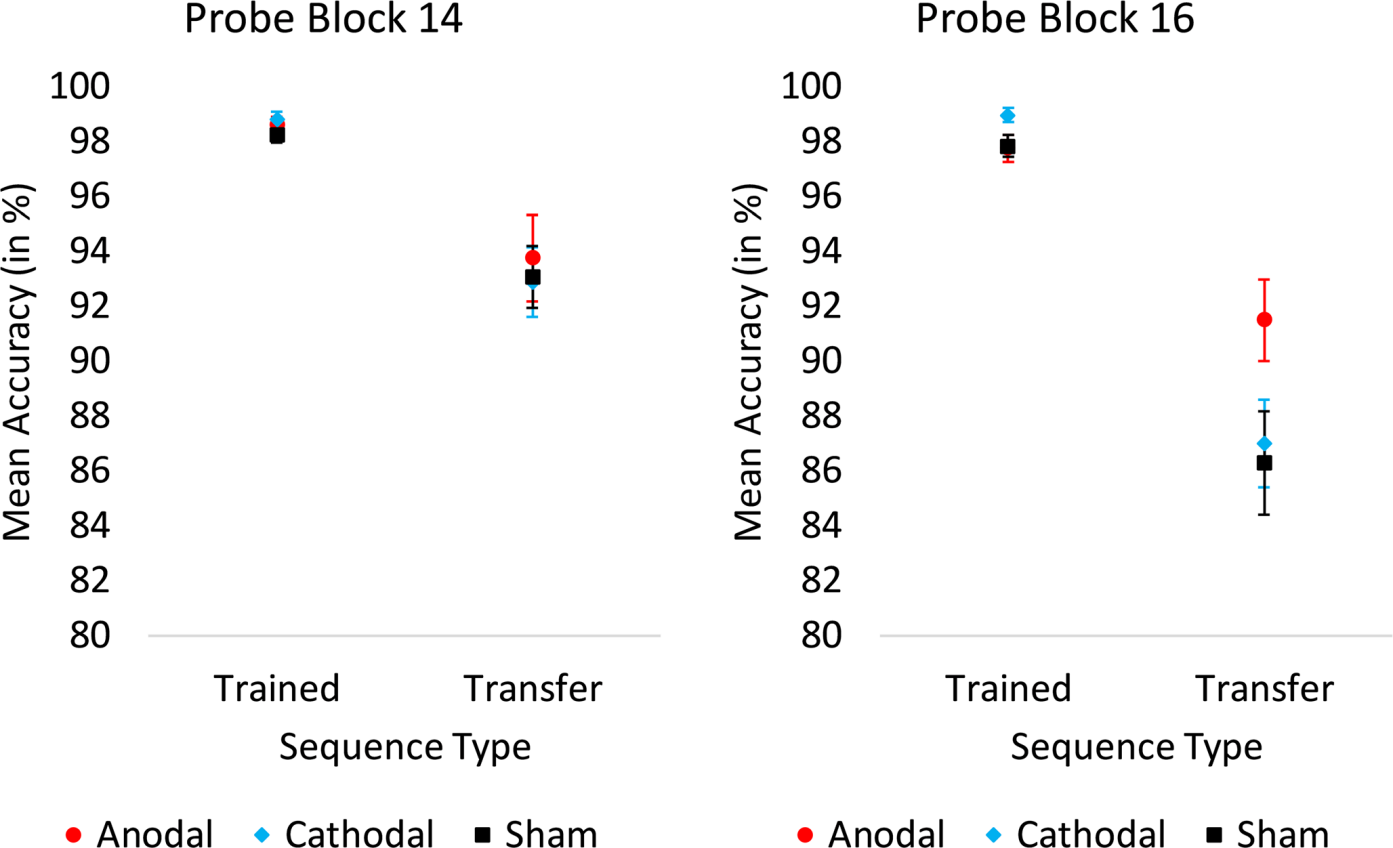
Mean accuracy (in percentage) as a function of stimulation group, sequence type, and probe block during test (at 24-h follow-up). Whereas there were no group differences in block 14, in block 16 there was selectively more accurate performance in the anodal stimulation group for the transfer sequence. Error bars represent standard error of the means

However, analysis of probe blocks 14 and 16 at follow-up did demonstrate a significant group × block × sequence type interaction (*F*[2,69] = 3.40, *p* = .039). This interaction was driven by the fact that there were no group differences in block 14 (see Fig. 4). In contrast, in block 16, the anodal group demonstrated lower ACC for the trained sequence (*M* = 97.74%, SD = 2.49%) as compared to the cathodal group (*M* = 98.96%, SD = 1.96%, *p* = .035) but not the sham group (*M* = 98.18%, SD = 2.01%, *p* = .877), with the latter almost differing significantly from each other (*p* = .050). Furthermore, in block 16, the anodal group demonstrated a higher ACC specifically for the transfer sequence (*M* = 91.49%, SD = 7.21%) as compared to the sham group (*M* = 86.28%, SD = 9.15%, *p* = .029) and trend-wise as compared to the cathodal group (*M* = 86.98%, SD = 7.80%, *p* = .058), with the latter two not differing significantly from each other (*p* = .767) (see Fig. 4).

## Discussion

The present study investigated the effects of cerebellar tDCS on motor response selection and sequence acquisition. In brief, the results indicate no group differences between cathodal and sham stimulations. However, there was impaired response selection under anodal as compared to cathodal and sham tDCS, as evidenced by an increase in overall RT during stimulation. This group difference became progressively smaller as the task progressed, but it reappeared at 24-h follow-up when participants performed the task without stimulation. Crucially, this difference was not pre-existing before intervention, as RT performance before stimulation did not differ between the groups. Overall, the results are consistent with the notion that cerebellar tDCS can affect motor response selection, possibly by modulating the inhibitory tone of the cerebellum over cortical motor areas.

The nature of performance impairment during anodal tDCS is consistent with a decline in motor response selection rather than impaired sequence learning. All three stimulation groups demonstrated a general practice effect during training, as evidenced by decreased RT as the task progressed. Moreover, all three stimulation groups demonstrated sequence-specific learning, evidenced by an increasing difference in RT between trained and transfer sequences in probe blocks. However, anodal tDCS was associated with increased RT during stimulation and, importantly, this effect did not depend on the specific SOC sequence (trained or transfer) being performed. This suggests anodal tDCS did not selectively hinder performance of the trained sequence; instead, it produced a non-specific delay in initiation of responses. This delay did decrease across training, which is consistent with the notion that cerebellar involvement in sequential motor performance and stimulus processing decreases over time [9, 23]. Accordingly, anodal stimulation of the cerebellum had less impact on response selection as training progressed.

However, at 24-h follow-up, the group that previously received anodal tDCS again demonstrated increased RT. This effect was restricted to probe blocks, in which both the well-trained SOC sequence as well as the less-practiced transfer sequence needed to be performed. Given that the effect of a single 20-min bout of tDCS on cortical excitability is supposedly of short duration (approximately 1 h) [14], it is unlikely that this long-term impact on performance is due to a persisting change of cerebellar or M1 excitability. Instead, although anodal tDCS had only a non-specific effect on RT during training, the particular impairment of performance at follow-up when the trained and transfer sequence had to be performed in the same experimental block tentatively indicates that the stimulation may have impaired consolidation/retention of the trained sequence structure. Impaired consolidation of the trained sequence would presumably worsen performance of this sequence in probe blocks, where it needed to be performed in close temporal succession to the transfer sequence. In line with this finding, the anodal group demonstrated higher RT and lower ACC for the trained sequence but higher ACC for the transfer sequence in the final probe block at follow-up. Assuming that the trained SOC sequence was indeed less consolidated due to anodal stimulation, this may have resulted in less interference from the trained sequence while performing the transfer sequence. Consequently, performance of the transfer sequence was more accurate.

This interpretation is consistent with the idea that motor memory consolidation can be inferred from its susceptibility to interference [62]. Such consolidation is thought to result from synaptic plasticity in M1, whereby long-term potentiation processes allow for a reorganization of M1 [63, 64]. Stronger consolidation is then inferred from less interference and a stronger retention of performance improvement across time, whereas weaker consolidation would produce the opposite pattern. In the present study, it is conceivable that anodal cerebellar tDCS may have interfered with sequence consolidation at one or more of different stages of learning. First, it is possible that an impairment of response selection processes (i.e., the observed increase in RT on day 1) may have interfered with consolidation already at the acquisition stage. That is, the execution of the sequence was hindered and thus the retention of this sequence was consequently impaired. Secondly, it is possible that reduced M1 excitability (via a strengthening of CBI) may have interfered with the long-term potentiation processes that underlie consolidation of the trained sequence. Indeed, sequence learning is typically associated with an increase in M1 excitability [33] and increased CBI may thus have interfered with this process.

The present finding of increased RT under anodal cerebellar tDCS converges with a previous report of delayed initiation of muscle activity under this type of stimulation [28]. From a theoretical perspective, the result also fits with previous studies applying anodal tDCS directly over M1. Whereas anodal stimulation of the cerebellum presumably decreases M1 excitability via strengthened CBI that is mediated by the dentate-thalamo-cortical pathway [30, 31], direct application of anodal tDCS over M1 should increase M1 excitability [4]. As expected, anodal tDCS directly over M1 produced opposite behavioral results from those observed in the present study: previous studies have reported enhanced response selection as evidenced by faster responses in an SRTT [25, 65, 66]. Although these studies varied in their methods of analysis, they indicate that increasing excitability of M1 can facilitate overall response selection and motor sequence learning. Taken together with the findings from the present study, convergent evidence indicates that increasing excitability of M1 by directly applying anodal tDCS to this region facilitates response selection, whereas indirectly decreasing its excitability by applying anodal tDCS to the cerebellum produces the opposite behavioral result.

### Future Directions

The present study has also raised important questions that need to be addressed in future work. In this regard, it should be mentioned that the present findings contrast with previous reports on anodal tDCS over the cerebellum and SRTT performance [24, 25], which demonstrated enhanced rather than impaired sequential performance. Of potential relevance is the fact that one of the studies used a symbolic rather than spatial stimulus–response mapping [25]. Previous studies indicate that performance improvements on the SRTT depend in large part on the learning of stimulus–response associations [11, 12]. It is therefore possible that enhanced SRTT performance under cerebellar tDCS was driven by a facilitated acquisition of the abstract stimulus–response associations, leaving it unclear whether the same performance enhancement is observable with spatial stimulus–response associations. This issue could be explored in future studies that aim to replicate the present experimental design using a symbolic instead of spatial stimulus–response mapping. The other study that demonstrated enhanced performance with anodal tDCS over the cerebellum based this conclusion on the comparison with a sham stimulation group that did not demonstrate any sequence learning [24]. Considering that SRTT performance typically does demonstrate a sequence learning pattern [13, 26, 27], the effectiveness of cerebellar tDCS appeared to be driven by an unexpected lack of improvement *without* stimulation rather than enhanced performance *with* stimulation. In light of this heterogeneity of results, there is a strong need for systematic and independent replication of these previous and current findings.

It should also be noted that the SRT paradigm in the present study included probe blocks wherein 10 runs of the trained sequence were intermixed with 2 runs of an untrained transfer sequence [52]. This procedure allowed us to investigate whether performance changes were dependent on the trained sequence or instead reflected general motor improvement. However, this particular distribution of trials does lead to less statistical power for the transfer as compared to the trained sequence. While we adopted this particular frequency in order to prevent the transfer sequence from interfering too much with acquisition of the trained sequence (cf. [42]), it would be advantageous for future studies to find a suitable compromise whereby statistical power for the transfer sequence is increased without suppressing the subjects’ acquisition of the trained sequence.

Furthermore, the fact that cerebellar tDCS affected performance not only during stimulation but also at later follow-up highlights the importance of incorporating delayed testing in SRTT paradigms and tDCS studies. Although currently more of an exception rather than a rule in SRTT research, delayed testing allows for a distinction between what has been referred to as “performance” versus “learning” [37]. The former is reflected in change in motor behavior *during* practice, whereas the latter is reflected in the retention of this change *after* practice. In particular for research on brain stimulation, the consolidation and retention of motor behavior is of most interest with regard to the development of potential enhancement and rehabilitation regimes. As such, future work should aim to include delayed testing of motor practice, perhaps even at longer and multiple intervals, in order to better distinguish between transient effects of tDCS on performance and longer-lasting effects mediated by a modulation of learning.

It should also be noted that the present study found behavioral effects of tDCS over the cerebellum exclusively for anodal stimulation, whereas it was previously shown that cathodal tDCS over the cerebellum decreases CBI [32] and therefore could potentially produce opposite behavioral results as those observed with anodal stimulation. Notably, the previously reported effect of cathodal tDCS on CBI was obtained with a current intensity of 2 mA, whereas in the present study we used the lower intensity of 1 mA. Hence, we speculate that the stimulation intensity used in the present study was not sufficient for behavioral effects of cathodal stimulation to become apparent. As such, future studies should investigate whether the effect of cathodal tDCS over the cerebellum is dose dependent and if at a higher current intensity it indeed produces opposite behavioral effects as those obtained with anodal stimulation.

Lastly, it should be noted that although the present study demonstrated an impairment of motor response selection in the SRTT under anodal cerebellar tDCS, the same form of stimulation has previously been observed to enhance performance particularly in paradigms tapping into motor adaptation [5, 19–22]. Although learning in the domains of motor adaption and motor sequences depend on shared neural structures [23], our results indicate that the effects of cerebellar tDCS are nevertheless task specific. As such, it is important for future work to assess the effect of cerebellar tDCS using a variety of experimental paradigms in order to elucidate the circumstances under which cerebellar tDCS might enhance or impair performance.

## Conclusions

In summary, the present study adds to a recently established body of literature by reporting on the effects of cerebellar tDCS on motor response selection and sequence acquisition. In brief, the results are consistent with the idea that cerebellar tDCS affects CBI, thereby modulating M1 excitability and the efficiency of response selection processes. Notably, the behavioral effect of anodal tDCS was not transient but persisted at 24-h follow-up, suggesting that stimulation did not only suppress immediate performance but also affected the retention of motor practice. This highlights the need for future work to include delayed testing, allowing for a discrimination between immediate changes in motor performance as compared to long-term effects on consolidation of practice benefits.
